# Flower-Like Plasma Cell Nuclei in Multiple Myeloma

**DOI:** 10.4274/tjh.galenos.2020.2020.0471

**Published:** 2021-06-01

**Authors:** Abibatou Sall, Moussa Seck, Diama Samb, Blaise Faye, Macoura Gadji, Saliou Diop, Awa Oumar Touré

**Affiliations:** 1 Dalal Jamm Hospital, Laboratory of Hematology, Dakar, Senegal; 2 Cheikh Anta Diop University-Hematology, Hematology Clinic Department, Dakar, Senegal; 3 Aristide Le Dantec Hospital-Laboratory of Hematology Pasteur Avenue, Dakar, Senegal

**Keywords:** Plasma cell, Morphologic abnormalities, Multiple myeloma

A 43-year-old Senegalese man with no known past medical history was referred to our hospital for asthenia and bone pain. The blood count showed moderate leukocytosis (12x10^9^/L), severe anemia (hemoglobin: 53 g/L), and a normal platelet count (315x10^9^/L), while the blood smear examination showed marked rouleaux formation. Protein electrophoresis showed a monoclonal gamma peak (82 g/L, Figure 1F). Immunofixation revealed a monoclonal immunoglobin G kappa band.

The diagnosis of multiple myeloma was confirmed by bone marrow aspiration. Giemsa-stained marrow smears showed hypercellularity with a large majority of very atypical plasma cells, namely medium-sized to large cells with nuclear abnormalities (budding: Figure 1A), flower-shaped nuclei that are quite uncommon in myeloma (Figures 1B-1E), and prominent nucleoli (Figure 1D, red arrow). Lymphoplasmacytic cells were also present, as well as several mitotic events (Figure 1D, black and blue arrows).

On flow cytometry the plasma cells expressed weak CD45, CD38, CD138, and CD56 (Figures 1G and 1H) and cytoplasmic kappa light chain. CD19, CD20, CD79a, and CD10 were negative. HIV, HBV, HCV, and HTLV-1 serology were negative. FISH was not available; thus, we could not calculate the Revised International Staging System score. However, the International Staging System result was stage III (β2 microglobulin = 5.9 mg/L).

Multiple myeloma is becoming more and more frequent in African populations. The key difference between African and Caucasian populations is the age of onset: 45-50 years in African populations and more than 60 years in Caucasians [1]. In addition, we note the presence of many poor prognosis factors in African patients resulting in earlier death. Delayed diagnosis and unavailability of new therapeutic agents and autografting could contribute to the poor outcome. However, genetic background and environmental factors could play a critical role and merit further studies [2].

## Figures and Tables

**Figure 1:**
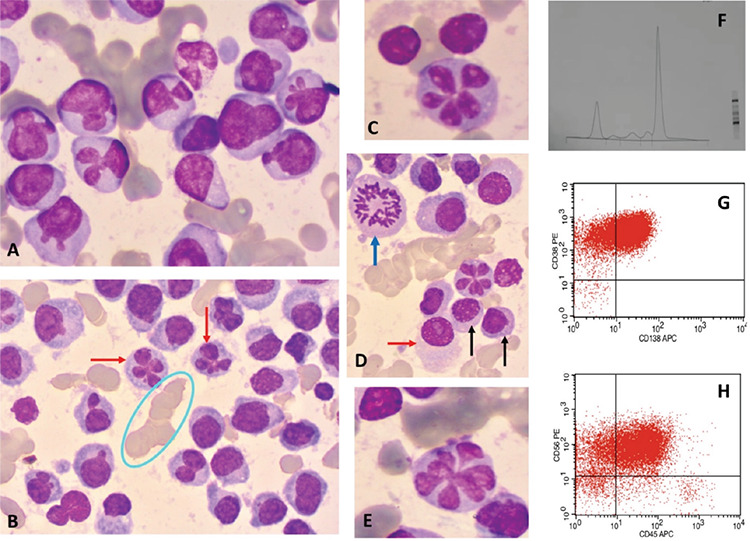
**A)** Plasma cells with dysmorphic nuclei. **B)** Flower-like nuclei (red arrow) and rouleaux (light blue circle). **C, E)** Flower-like nuclei. **D)** Mitotic event (blue arrow). Lymphoplasmacytic cells (black arrows). Red arrow: plasma cell with a prominent nucleolus. **F)** Monoclonal gamma globulin peak on protein electrophoresis. **G, H)** Flow cytometry showing CD38/138+, CD45^weak^, and CD56+.
